# Student Perceptions and Acceptance of Mobile Technology in an Undergraduate Nursing Program

**DOI:** 10.3390/healthcare5030035

**Published:** 2017-07-21

**Authors:** Tracy P. George, Claire DeCristofaro, Pamela F. Murphy, Archie Sims

**Affiliations:** 1 Department of Nursing, Francis Marion University, Florence, SC 29502, USA; 2 Department of Behavioral Sciences, College of Health, Human Services, and Science, Ashford University, San Diego, CA 92123, USA; claire.decristofaro@ashford.edu (C.D.); pamela.murphy@ashford.edu (P.F.M.); 3 Palmetto Health Tuomey, Sumter, SC 29150, USA; Archie.Sims@palmettohealth.org

**Keywords:** smartphones, technology, apps, nursing preventive care, nursing leadership, population health management, health information literacy, health equity

## Abstract

Mobile technology allows healthcare students to access current evidence-based resources. The purpose of this study was to evaluate the student experience of implementing point-of-care (POC) smartphone applications in a first-semester undergraduate nursing program. Teaching methods included using case studies in the laboratory to familiarize students with the apps. At community screening sites, evidence-based guidelines were referenced when students discussed screening results with patients. Surveys were administered prior to implementing this innovation and after the students utilized the apps in direct patient interactions. Survey results were analyzed to evaluate student perceptions and acceptance of mobile technology. Students felt that healthcare smartphone apps were a helpful and convenient way to obtain evidence-based clinical information pertinent to direct care settings. Over 90% of students planned to continue using healthcare smartphone apps. In conclusion, healthcare smartphone apps are a way for students to become comfortable accessing evidence-based clinical resources. It is important to encourage students to use these resources early in the curriculum. Community screenings are an independent health promotion activity which assists in the attainment of health equity and fosters nursing leadership.

## 1. Introduction

Smartphones are portable and are updated frequently, as compared to textbooks which become obsolete quickly [1]. In order to promote patient safety and high quality nursing care, nurses need access to current clinical resources [2]. Grabowsky (2015) found that 80% of nurse practitioners in clinical practice use smartphone applications (apps) for pharmacology information [3]. Minority and lower-income nursing students are less likely to have internet access at home, but they are likely to own smartphones [4]. In the United States, 72% of Americans own smartphones [5]. Most nursing students are adept at the use of smartphones and find them to be convenient in the clinical setting for decision-making [6]. Smartphone apps are a way for nursing students to utilize point of care (POC) resources to promote patient safety and evidence-based care.

Smartphone apps have been used extensively in the clinical setting [7]. Doyle, Garrett, and Currie (2014) found that mobile devices are utilized to obtain medication information and for clinical logs, peer support, and communication between faculty and students [8]. Point of care access to clinical practice guidelines, pharmacology resources, videos, and podcasts promotes patient safety [9]. In a study of undergraduate nursing students, they felt that smartphone apps were beneficial in the clinical setting [10]. Smartphone apps can also provide useful information on recommended evidence-based screenings [9].

However, some nurses in the clinical setting may view the use of smartphones negatively. In a qualitative study with 13 nursing students and five nurse managers, nursing students reported that smartphones are helpful when making clinical decisions, but nurse managers viewed smartphones use as being potentially unprofessional and unethical [6]. It is important for nursing faculty members to educate nurse managers that smartphones are an evidence-based resource for nursing students and nurses.

There is a need for more interactive activities in the classroom and laboratory setting using smartphones [7]. Smartphones can be used in the classroom setting to encourage active learning through case studies, research on nursing topics, and small group work [11]. Smartphones can also be used as a clicker device in the classroom to encourage student engagement in the content [9]. In order to prepare students for clinical practice, informatics and health care technology should be integrated into a variety of nursing courses, including pharmacology, health assessment, pathophysiology and clinical courses [12].

Faculty training is important when adopting technology in order to help overcome faculty resistance to integrating this technology into the classroom, clinical or laboratory setting [7]. In an advanced health assessment course, faculty training with the devices and software was important to the success of an educational innovation, which included case studies using blended POC Software (Lexicomp^®^) with high-fidelity simulation mannequins [13]. Use of case studies that require smartphone apps can help familiarize both faculty and students with this technology, facilitating acceptance and use in direct patient care [14].

There are potential barriers to the implementation of smartphone technology. The price of smartphones and apps can be a barrier to their use in nursing education [7]. However, smartphones are becoming more prevalent today, and many free apps are available. In addition, some universities purchase databases which may be accessed using point of care technology in the classroom or clinical setting. In one study, the small screen size, difficulty with internet connections, lack of student comfort with technology, and learning resources that were incompatible with the iPods negatively impacted the use of handheld mobile devices [15]. Although smartphones can serve as useful clinical references, they may not be ideal for detailed research on clinical topics. In a randomized controlled trial with 120 medical students in a clinical setting, tablets and smartphones were valued for their mobility. However, students felt that computers were more useful for doing more extensive literature searches for evidence-based medicine [16]. These barriers may be overcome in future with advances in mobile health technology. Such advances may include more robust apps as well as hardware attachments for smartphones, which may result in improved practice and student engagement in the content [17]. For example, an otoscope adapter can transform a smartphone into an otoscope, permitting increased skills practice.

The purpose of this study was to incorporate POC technology in both the laboratory and field settings for an undergraduate nursing health assessment course. By including this learning activity in the first semester, students are introduced to the concept of real-time, current, and evidence-based resources that can support their nursing practice. This learning activity had been piloted previously by the authors in the same course and curriculum level. This report provides data on student perceptions on the application of this technology in the setting of community health screenings.

## 2. Materials and Methods

This study was an anonymous survey of pre-licensure first-semester Bachelor of Science (BSN) students at a public, rural, four-year university in the Southeast United States. This project was a follow-up to a prior non-data driven study with smartphones and first semester nursing students [14]. Qualitative data were obtained through content analysis of students’ responses to six open-ended survey questions, and authors TPG and CD coded the survey questions to identify and determine repetitive themes; agreement was reached on any differences. Surveyed subjects were students enrolled in the course in fall 2016 semester, and who participated in learning activities in the laboratory setting and community screenings. These apps included a body mass index (BMI) calculator [18,19] and the electronic preventive service selector (ePSS) apps from the Agency for Healthcare Research & Quality [20]. The ePSS app uses patient age, sex, smoking status, and sexual activity to provide individualized health screening recommendations based on current United States Preventive Services Task Force guidelines [20]. The Body Mass Index (BMI) app calculates BMI for adults [18,19]. All students in the class already owned smartphones, and their personal devices were used for this project. Both apps were and are available free of charge, and work cross-platform on Android and iPhone devices.

Survey data included demographic information and questions designed to assess their perception of ability, learning and motivation prior to and after the use of two free smartphone apps. The following survey questions were used in this study (see Table 1).

In week one, students downloaded and practiced with the two apps with faculty guidance in the laboratory setting. All students in the course owned a smartphone for their personal use, so this was not a barrier to the project. In weeks two and three, students completed interactive case studies requiring smartphone apps, with instructor facilitation. After this preparation, students participated in three hours of community screenings as part of the course requirements, starting in week four of the course. In addition to using the smartphone apps for BMI and preventive screening recommendations, each patient had blood pressure, blood glucose and cholesterol testing. Each screening participant received an educational packet along with their results, which included evidence based information on these biomarkers from the Joslin Diabetes Center [21], the Centers for Disease Control [22,23], and the National Heart, Lung and Blood Institute [24] of the National Institutes of Health (NIH), and AHRQ [25,26].

All subjects gave their written informed consent for inclusion before they participated in the study. The study was conducted in accordance with the Declaration of Helsinki, and the protocol was approved by the Institutional Review Board of Francis Marion University (project identification code George-03-14-2016-002, approved 14 March 2016). Students were advised that participation was not required, it was anonymous, and participation would not impact grades. Qualitative data on the post-survey was obtained through content analysis of six questions. The researchers coded the open-ended survey questions to identify and determine the categories and repetitive themes found. Any differences were discussed and consensus was reached on the themes. One author (TPG) was the instructor for the course, but surveys were administered by non-instructional staff.

## 3. Results

A total of 71 surveys were completed, out of 78 possible students, for a response rate of 91%. Respondents were 84.5% female (60/71), 15.5% male (11/71); 81.7% (58/71) White/Caucasian, 16.9% (12/71) Black/African-American, 1% other (1/71); 88.7% (63/71) age 18–25 years old, 8.5% (6/71) age 26–35 years old, 1.4% (1/71) age 36–45 years old, and 1.4% (1/71) age 46–55 years old (See Table 2).

The following themes emerged from content analysis of the survey questions. Some comments supported multiple themes (see Table 3).

Theme 1: Communication and patient interaction. Students felt that this learning activity benefited them by improving their comfort level when taking patient histories and providing patient education. One student said there was a “better interaction with patients and techniques” and another stated “I developed better social skills in a real-life setting”. Communication skills were also felt to be enhanced. Student comments included: “how to communicate information to multiple patients, ask the right questions, become comfortable during the appointment, interact naturally”.

Theme 2: Cultural competence. Of importance to future nursing interactions with the area’s patient population, a student commented, “I learned how to communicate with people of different races and cultural backgrounds” and another stated, “how to deal with all kinds of people and how to have different approaches towards people”. An interesting comment was, “the patients aren’t scary”.

Theme 3: Patient education skills. Students were required to provide individualized patient education based on the ePSS app results. Comments included: “how to talk to patients about their results from blood glucose and cholesterol results” and “I was familiar with diabetes management and prevention; however this screening has enhanced my ability in skills in dealing with this disease”.

Theme 4: Knowledge base and application. Students felt they were able to translate didactic knowledge to real-world application. Comments include: “understanding how to implement what we learned in class”, “practice what we learned in lab”, “applying what we learned in class to real situations”, and “patient interaction and knowledge of the material”.

Theme 5: Nursing role. Comments included “it got me over my first patient anxiety”, “I feel more comfortable talking and having a patient/nurse interaction”, and “Confidence, talking to real patients, practice asking personal questions”.

Theme 6: Perception of smartphone apps. Overall, over 90% of students plan to continue using smartphone apps in their nursing practice. Students reported finding the apps “helpful”, “useful”, “I think they are convenient, it is nice to have all the information you need at your fingertips”, “I think they are very helpful and a great advancement in nursing”, “very quick and easy to use as a resource”, and “they are very useful and meaningful, being that my peers and I are dependent on technology and smartphones”.

Theme 7: Patient acceptance: students reported positive feedback and acceptance of the apps from patients. One nursing student stated that patients “were impressed that I was able to obtain information that quickly and accurately”, and “patients seemed comfortable with apps”.

## 4. Discussion

Students already use smartphones for communication and entertainment. Smartphone apps provide a convenient way for students to obtain updated healthcare information in the laboratory, community, and clinical settings. Students view apps as more portable than textbooks, and they are updated frequently as guidelines change. Many clinical apps are free, so there may not be costs incurred by students. The implementation of apps early in the nursing curriculum encourages active learning and the use of current, evidence-based resources when providing nursing care. When students begin to use apps during the first semester of the nursing program, they may be more likely to continue to use apps they progress in the program.

Themes identified by content analysis included communication and patient interaction, cultural competence, patient education skills, knowledge base and application, the nursing role, student perception of smartphone apps, and patient acceptance of smartphone apps. Overall, use of the smartphone apps was viewed as a positive activity, from students and screening participants alike. Of interest, although there may be a perception by the general public that the elderly are resistant to use of technology, these results agree with recent findings that this population actually are very accepting of the use of POC technology in healthcare [27]. The vast majority of students, as well as the nursing preceptors involved in the screening events, plan to continue to use smartphone apps in their nursing practice, validating the positive nature of their experience with POC technology.

The apps used at the community screenings allowed students to discuss evidence-based screening guidelines and provide accurate BMI results to patients. Community nurses who observed our students using the apps and evidence-based patient education materials commented that they intended to implement such technology in their own practice. Through participation in community screenings, nurses demonstrate leadership in the attainment of health equity through health promotion activities. Providing nursing students with training on POC technology and encouraging them to incorporate it into their nursing practice supports the delivery of patient-centered care. These students will be better prepared to provide current and evidence-based practice in settings that may be nontraditional, such as community screenings [9,14]. This will result in a more holistic approach to care.

## 5. Conclusions

Limitations to the current study includes a small sample size of survey subjects, all at one grade level in one nursing program over one semester. There were system issues in various aspects of the project, including technological challenges (e.g., smartphone device failure, app failure) as well as external problems such as weather and transportation to the external screening site. Lastly, some screening sites had insufficient numbers of patients due to no-shows. Many students described feeling nervous during the patient screenings, and this may have impacted their ability to successfully execute the application of skills and knowledge. Lastly, our survey sample was mainly young, Caucasian females, which may have skewed responses.

This study evaluated student perceptions regarding the use of POC technology, including smartphone apps, both in the laboratory and in the setting of community patient screenings. The use of these apps reinforced with nursing students the need to use reliable real-time resources. Overall, this active learning opportunity supported the transition to the real world of clinical practice, encouraging the development of nursing leadership, supporting holistic care practices, and improving community health equity. This study demonstrates that the adoption of mobile technology is a useful means to teach the importance of safe patient care and use of evidence-based guidelines. An implication for nursing curricula is to consider the inclusion of this type of learning activity early in the program.

## Figures and Tables

**Table 1:** Survey Questions.

What skills have you developed from participating in the community screening activity?
What were your best and worst experiences in the community screening activity?
Was there anything that affected your performance at the community screening activity?
What are your thoughts on the use of smartphone apps in nursing practice?
Will you continue to use smartphone apps in your nursing education and nursing practice?
What types of responses from patients did you receive when you used apps to provide them with patient education?

**Table 2:** Demographic characteristics of subjects.

**Gender**	
Female	84.5% (*n* = 60)
Male	15.5% (*n* = 11)
**Race**	
Caucasian	81.7% (*n* = 58)
Black/African/American	16.9% (*n* = 12)
Other Race	1% (*n* = 1)
**Age**	
18–25 years of age	88.7% (*n* = 63)
26–35 years of age	8.5% (*n* =6)
36–45 years of age	1.4% (*n* = 1)
46–55 years of age	1.4% (*n* = 1)

Note: Total number of students surveyed: 71.

**Table 3:** Survey Results: Themes and Examples.

Theme 1: Communication and patient interaction	Students felt that this learning activity benefited them by improving their comfort level when taking patient histories and providing patient education. One student said there was a “better interaction with patients and techniques” and another stated “I developed better social skills in a real-life setting”. Communication skills were also felt to be enhanced. Student comments included: “how to communicate information to multiple patients, ask the right questions, become comfortable during the appointment, interact naturally”.
Theme 2: Cultural competence	Of importance to future nursing interactions with the area’s patient population, a student commented, “I learned how to communicate with people of different races and cultural backgrounds” and another stated, “how to deal with all kinds of people and how to have different approaches towards people”. An interesting comment was, “the patients aren’t scary”.
Theme 3: Patient education skills	Students were required to provide individualized patient education based on the ePSS app results. Comments included: “how to talk to patients about their results from blood glucose and cholesterol results” and “I was familiar with diabetes management and prevention; however this screening has enhanced my ability in skills in dealing with this disease”.
Theme 4: Knowledge base and application	Students felt they were able to translate didactic knowledge to real-world application. Comments include: “understanding how to implement what we learned in class”, “practice what we learned in lab”, “applying what we learned in class to real situations”, and “patient interaction and knowledge of the material”.
Theme 5: Nursing role	Students indicated that the screenings assisted them in embracing the nursing role. Comments included “it got me over my first patient anxiety”, “I feel more comfortable talking and having a patient/nurse interaction”, and “Confidence, talking to real patients, practice asking personal questions”.
Theme 6: Perception of smartphone apps	Overall, over 90% of students plan to continue using smartphone apps in their nursing practice. Students reported finding the apps “helpful”, “useful”, “I think they are convenient, it is nice to have all the information you need at your fingertips”, “I think they are very helpful and a great advancement in nursing”, “very quick and easy to use as a resource”, and “they are very useful and meaningful, being that my peers and I are dependent on technology and smartphones”.
Theme 7: Patient acceptance	Students reported positive feedback and acceptance of the apps from patients. One nursing student stated that patients “were impressed that I was able to obtain information that quickly and accurately”, and “patients seemed comfortable with apps”.
